# Global Intracoronary Infusion of Allogeneic Cardiosphere-Derived Cells Improves Ventricular Function and Stimulates Endogenous Myocyte Regeneration throughout the Heart in Swine with Hibernating Myocardium

**DOI:** 10.1371/journal.pone.0113009

**Published:** 2014-11-17

**Authors:** Gen Suzuki, Brian R. Weil, Merced M. Leiker, Amanda E. Ribbeck, Rebeccah F. Young, Thomas R. Cimato, John M. Canty

**Affiliations:** 1 Division of Cardiovascular Medicine, Veterans Affairs Western New York Health Care System, Buffalo, New York, United States of America; 2 Department of Medicine, Division of Cardiovascular Medicine, University at Buffalo, Buffalo, New York, United States of America; 3 Department of Physiology & Biophysics, University at Buffalo, Buffalo, New York, United States of America; 4 Department of Biomedical Engineering, University at Buffalo, Buffalo, New York, United States of America; 5 The Clinical and Translational Research Center, University at Buffalo, Buffalo, New York, United States of America; Georgia Regents University, United States of America

## Abstract

**Background:**

Cardiosphere-derived cells (CDCs) improve ventricular function and reduce fibrotic volume when administered via an infarct-related artery using the “stop-flow” technique. Unfortunately, myocyte loss and dysfunction occur globally in many patients with ischemic and non-ischemic cardiomyopathy, necessitating an approach to distribute CDCs throughout the entire heart. We therefore determined whether global intracoronary infusion of CDCs under continuous flow improves contractile function and stimulates new myocyte formation.

**Methods and Results:**

Swine with hibernating myocardium from a chronic LAD occlusion were studied 3-months after instrumentation (n = 25). CDCs isolated from myocardial biopsies were infused into each major coronary artery (∼33×10^6^ icCDCs). Global icCDC infusion was safe and while ∼3% of injected CDCs were retained, they did not affect ventricular function or myocyte proliferation in normal animals. In contrast, four-weeks after icCDCs were administered to animals with hibernating myocardium, %LADWT increased from 23±6 to 51±5% (p<0.01). In diseased hearts, myocyte proliferation (phospho-histone-H3) increased in hibernating and remote regions with a concomitant increase in myocyte nuclear density. These effects were accompanied by reductions in myocyte diameter consistent with new myocyte formation. Only rare myocytes arose from sex-mismatched donor CDCs.

**Conclusions:**

Global icCDC infusion under continuous flow is feasible and improves contractile function, regresses myocyte cellular hypertrophy and increases myocyte proliferation in diseased but not normal hearts. New myocytes arising via differentiation of injected cells are rare, implicating stimulation of endogenous myocyte regeneration as the primary mechanism of repair.

## Introduction

A large number of preclinical studies have demonstrated the ability of diverse adult stem cell formulations to prevent post-infarction remodeling [1]. Stimulating resident progenitor cells or administering cell preparations that include exogenous cardiac stem cells may be particularly effective approaches to elicit cardiac repair. Although several stem and progenitor cell populations have been identified, there is compelling evidence that the reparative potential of these cells is maximized when delivered as a heterogeneous mixture of heart-derived subpopulations[2]–[4]. This notion has been supported by the encouraging results of studies utilizing cardiosphere-derived cells (CDCs), a population of cardiac stromal cells derived from myocardial biopsies that fulfill the criteria for cardiac progenitor cells and have recently been shown to reduce scar mass and increase viable myocardium in patients with left ventricular dysfunction after myocardial infarction [5].

At this stage of therapeutic development, most basic and clinical studies have focused on infusing stem cells down the infarct-related artery with the “stop-flow” technique or injecting them into the peri-infarct tissue in an attempt to replace scar with newly regenerated myocardial tissue. While these approaches may effect repair in the infarcted region, viable dysfunctional myocardium without fibrosis may also be an important target for cardiac repair. Viable dysfunctional regions develop as a consequence of repetitive ischemia as in hibernating myocardium where there is regional myocyte loss and compensatory myocyte hypertrophy [6]. In addition, viable dysfunctional myocardium can also develop in regions that are normally perfused and remote from a large dysfunctional region (viable or infarcted) due to apoptosis-induced myocyte loss and left ventricular remodeling [7], [8]. Importantly, despite revascularization procedures, these viable dysfunctional regions comprise a much larger percent of the heart in patients with advanced ischemic cardiomyopathy as infarct volume only averages ∼20% of the left ventricle (LV) [9]. Thus, regenerating myocytes globally, including areas of dysfunctional myocardium without scar, may be required to reverse deleterious LV remodeling and optimally improve LV function.

To determine the extent that the reparative actions of CDCs may be independent of scar replacement, we administered cells via intracoronary infusion to the entire heart under continuous flow in a swine model of hibernating myocardium. Myocyte proliferation was assessed in hibernating as well as normally-perfused remote regions and sex-mismatched allogeneic CDCs were used to quantify myocytes arising directly from the injected cells. Our results demonstrate that global intracoronary infusion of CDCs without using the “stop-flow” technique improves myocardial function. These effects are primarily related to actions on endogenous myocytes in hibernating regions as well as normally-perfused remote myocardium with only rare myocytes arising from donor cells.

## Methods

### Ethics Statement

**Figure 1:**
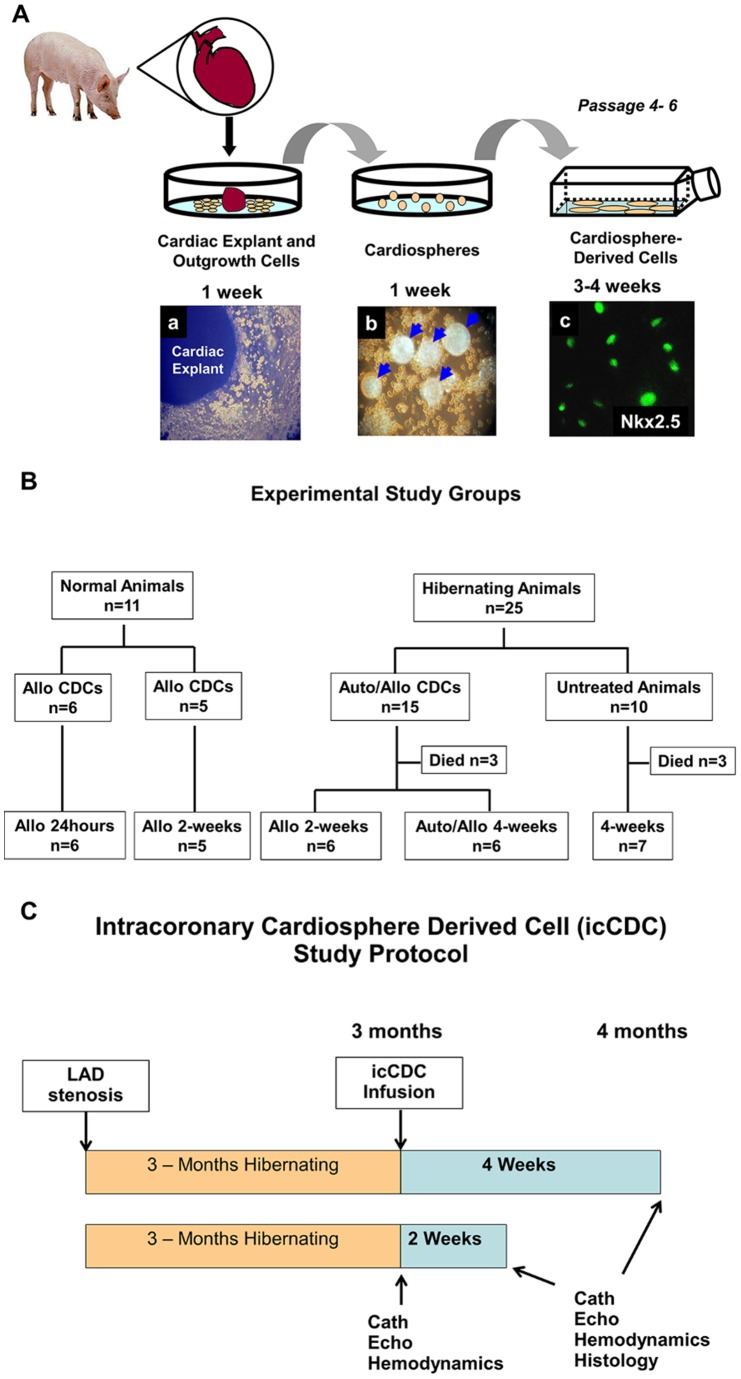
CDC Isolation, Summary of Experimental Study Groups, and Study Protocol Timeline. **A.)** Myocardial needle biopsies were obtained from the LV free wall at the time of initial instrumentation in juvenile pigs with hibernating myocardium. The tissue fragments were cultured as “explants” on dishes coated with fibronectin. After one week, a layer of stromal-like cells started surrounding the explants (a). A population of small, round and phase-bright cells developed over this layer that subsequently formed cardiospheres (b). These cells were harvested and began forming cardiosphere-derived cells which are stained in green with the cardiac transcription factor Nkx2.5 (c). Expansion of CDCs to obtain a total injected volume >30 million CDCs typically required six passages in culture. **B.)** Experimental data was acquired from 36 animals. Eleven pigs were normal and used to exclude myocardial necrosis after infusing icCDCs as well as assess CDC retention and the effects of icCDCs on myocyte proliferation in the normal heart. Twenty-five animals had viable dysfunctional myocardium (hibernating animals) and were studied 3-months after the development of a chronic LAD occlusion and the development of chronic hibernating myocardium. Of the animals with hibernating myocardium, 15 were randomized to receive intracoronary CDC infusion and 10 served as untreated controls. Follow-up in animals with icCDCs was at either 24 weeks after icCDC infusion. Sudden cardiac death from lethal ventricular arrhythmias [22] occurred before the final study was completed in six animals with hibernating myocardium (3 in untreated and 3 in icCDC treated). These rates are similar to those previously published. Auto – autologous; Allo – allogeneic. **C.)** Juvenile swine were instrumented with a Delrin LAD stenosis to produce hibernating myocardium with serial physiological studies beginning at 3-months. At that time, a baseline closed-chest study was conducted to assess myocardial function with echocardiography (echo), myocardial perfusion with microspheres at rest and vasodilation, hemodynamics and coronary angiography (Cath). Subsequently, intracoronary CDCs were infused throughout the heart in 15 animals and 10 served as untreated controls. One group of animals underwent repeat study and tissue harvesting after 2-weeks of treatment and the second group was evaluated 4-weeks after icCDC treatment.

All procedures and protocols conformed to institutional guidelines for the care and use of animals in research and were approved by the University at Buffalo Institutional Animal Care and Use Committee (Protocol #MED02011Y). All studies were performed under either isoflurane or propofol anesthesia as described below, and all efforts were made to minimize suffering. **Figure 1** outlines the experimental groups and steps in the isolation of CDCs described below.

### Cultivation, Expansion and In Vitro Characterization of CDCs

CDCs were cultivated using the techniques described by Smith et al [2] (**Figure 1A**). LV tissue specimens were obtained by needle biopsies (2–5 biopsies from the LV basal free wall, 20–50 mg total) and cut into 1–2 mm pieces [2], [10]. After gross connective tissue was removed from fragments, they were washed and partially enzymatically digested in a solution of type IV collagenase for 60 minutes at 37 degrees. Tissue fragments were cultured as “explants” on dishes coated with fibronectin. After ∼8 days, a layer of stromal-like cells arose from and surrounded the explants. Over this layer a population of small, round, phase-bright cells migrated. Once confluent, the cells surrounding the explants were harvested by gentle enzymatic digestion. These cardiosphere-forming cells were seeded at 2 to 3×10^4^ cells/mL on poly-D-lysine-coated dishes in cardiosphere medium (20% heat-inactivated fetal calf serum, gentamicin 50 µg/ml, 2 mmol/L L-glutamine, and 0.1 mmol/L 2-mercaptoethanol in Iscove’s modified Dulbecco medium). Cardiospheres formed after 4–10 days in culture, detached from the tissue culture surface, and began to slowly grow in suspension. When sufficient in size and number, free-floating cardiospheres were harvested by aspirating them along with media. Cells that remained adherent to the poly-D-lysine-coated dishes were discarded. Detached cardiospheres were then plated on fibronectin-coated flasks where they attached to the culture surface and formed monolayers of “Cardiosphere-Derived Cells” (CDCs) [2]. CDCs were subsequently passaged by trypsinization and splitting at a 1∶2 ratio. Up to 100 million CDCs developed within 4–6 weeks of the time that the original cardiac biopsies were obtained. Cells were characterized by flow cytometry and immunohistochemistry with hematopoietic (CD45, cKit, CD133), mesenchymal (CD90, CD105) and cardiac (GATA4, Nkx2.5, cTnT, and cTnI) markers.

Prior to administration, cell suspensions were filtered through a 30–100 µm pore filter to circumvent administering cell aggregates (MACS pre-separation filters, Miltenyi Biotec) and suspended in heparinized HBSS solution (3000 U heparin in 30 ml in total) for intracoronary infusion. Approximately 10–15×10^6^ cells were infused into each of the three proximal coronary arteries including the stump of the occluded left anterior descending artery (LAD). Thus, cells were administered to the entire heart with the hibernating LAD region receiving cells through the stenotic LAD and/or collateral vessels that typically develop in this model. The cell suspensions were each slowly infused over 10-minutes with no untoward hemodynamic changes or electrocardiographic evidence of myocardial ischemia.

### In Vivo Studies of Intracoronary CDC (icCDC) Infusion in Swine

The effects of intracoronary CDC infusion on flow, function and myocyte proliferation were assessed in normal animals as well as a series of animals with viable dysfunctional hibernating myocardium. Experimental groups and samples sizes are summarized in **Figure 1B**. All animals were in good health at the time of study and the specific protocols performed in each group are summarized below.

#### Intracoronary CDC Infusion in Normal Swine (n = 11)

To assess the safety of infusing allogeneic intracoronary CDCs throughout the entire heart, the acute electrocardiographic, hemodynamic and functional effects of global icCDC infusion were assessed in normal animals**.** In six animals, serial echocardiography and ST-T changes were assessed by Holter monitoring and hearts were excised after 24-hours to evaluate postmortem necrosis by pathology and TTC staining. We measured average CDC size immediately prior to cell infusion with a hemocytometer. To exclude minor injury beyond the detection of TTC and pathology, serum cardiac TnI (pig cTnI ELISA Kit, Life Diagnostics) was assessed at baseline, 2 hours and 24 hours after injection. Less than 0.04 ng/ml of serum cTnI was considered normal. Regional and global function was assessed with echocardiography at baseline and 2-hours after icCDC infusion. In an additional 5 pigs, follow-up was carried out for 2-weeks to assess late retention and the effects of icCDC infusion on myocyte nuclear density, morphometry and immunohistochemistry in the completely normal heart. TTC staining was also performed to exclude necrosis.

#### Intracoronary CDC Infusion in Swine with Hibernating Myocardium (n = 25)

In order to study the effects of icCDCs on cardiac myocyte function in a fashion that was independent of myocardial scar a porcine model of viable dysfunctional hibernating myocardium was produced as previously described [11]. Briefly, juvenile pigs were sedated (Telazol 100 mg/ml/xylazine 100 mg/ml, 0.022 mg/kg i.m.), intubated and ventilated with a 0.5–2% isoflurane-oxygen mixture. Through a limited pericardiotomy, the proximal LAD was instrumented with a Delrin occluder (1.5 mm). Antibiotics (cefazolin, 25 mg/kg and gentamicin, 3 mg/kg i.m.) were given 1-hour before surgery and repeated after closing the chest. Analgesia included an intercostal nerve block (0.5% Marcaine) and intramuscular doses of butorphanol (2.2 mg/kg q6h) and flunixin (1–2 mg/kg q.d.). Pigs with hibernating myocardium underwent initial studies 3-months after instrumentation. We have previously demonstrated that reductions in resting flow, function and flow reserve develop in this model after 3-months and remain unchanged between 3- and 5-months after instrumentation producing a stable model of ischemic LV dysfunction [11], [12]. In contrast to models of total coronary occlusion, histological and TTC evidence of infarction is absent in this model.

#### Serial Physiological Studies [12]

The timeline of physiological studies is summarized in **Figure 1C**. Animals with chronic hibernating myocardium underwent an initial baseline physiological study 3-months after initial instrumentation to establish the presence of contractile dysfunction at rest. Sedation was initiated with a Telazol (100 mg/ml)/xylazine (100 mg/ml) mixture (0.037 ml/kg i.m.) and maintained with propofol (5–10 mg/kg/hr i.v.). Under sterile conditions, a 6-Fr introducer was inserted into the left brachial artery. A 5F Millar micromanometer was placed into the LV apex with the lumen used for microsphere injection. The introducer side port was used to monitor aortic pressure and provide a reference blood withdrawal for microspheres. Animals were heparinized (100 U/kg), and hemodynamics allowed to equilibrate for at least 30-minutes. Regional wall-thickening was assessed using off-axis M-Mode echocardiography (GE Vivid 7) employing a right parasternal approach. All hibernating pigs had contractile dysfunction in the distribution of the anterior wall supplied by the LAD. To quantify this, systolic wall-thickening (ΔWT = ESWT-EDWT; %WT = ΔWT/EDWTx100) was measured in the dysfunctional LAD region as well as in normally-perfused remote regions (posterior wall) of the same heart. All measurements were calculated from echocardiographic dimensions using ASE criteria. After baseline measurements, myocardial perfusion was assessed with microspheres at rest and following pharmacological vasodilation using adenosine (0.9 mg/kg/min iv) while phenylephrine was infused and titrated to maintain mean blood pressure at ∼100 mmHg. Subsequently, animals received icCDCs (n = 15) or were untreated (n = 10). Since initial results (n = 6) demonstrated comparable effects of autologous and allogeneic icCDCs, allogeneic icCDCs with cyclosporine A immunosuppression (5 mg/kg/day p.o., Watson Pharma) were used for the majority of experiments (20/23 icCDC-treated animals). At the end of each study, the catheters were removed and pigs were allowed to recover and returned to the animal housing facility. Two- or four-weeks later, the pigs were brought back to the laboratory for a second physiological study which was performed in a fashion similar to the initial baseline protocol. Once measurements were completed, animals were euthanized under general anesthesia. The LV was rapidly excised, weighed and sectioned into 1-cm rings parallel to the AV groove from apex to base. Thin rings above each major ring were incubated in TTC to assess infarction. Additional tissue samples were taken for quantifying microsphere flow and histology.

#### Microsphere Flow Measurements [13]

Regional perfusion was assessed using 15 µm microspheres labeled with fluorescing dyes [14], [15]. Approximately 3×10^6^ microspheres were injected into the LV while a reference sample was withdrawn at 6 ml/min for 90-seconds. At the end of the study, samples were taken from a mid-ventricular ring and divided into twelve circumferential wedges, each of which was cut into 3 transmural layers. Fluorescent dyes were extracted using standard techniques and fluorescence quantified at selected excitation wavelengths. In each animal the circumferential flow distribution during adenosine was analyzed to identify the hibernating risk region as compared to normal regions where flow increased 4–6 fold. From these data, we determined the weighted average flow from samples in the central hypoperfused region (hibernating LAD) or normally-perfused remote region. Samples with intermediate vasodilated flows were considered border regions and excluded from analysis. Using these data, we also assessed relative and absolute coronary flow reserve in animals receiving icCDCs vs. untreated swine with hibernating myocardium using. Relative flow reserve was determined by dividing the flow in LAD regions by the corresponding average full-thickness value from normal myocardium. Absolute coronary flow reserve was assessed by comparing flow in each region during vasodilation to the corresponding values at rest.

### Myocardial Histopathology and Flow Cytometry of Circulating Progenitor Cells

Quantitative immunohistochemistry and morphometry were evaluated in icCDC-treated and untreated animals with hibernating myocardium as previously described in detail and summarized below [12], [16].

#### Myocyte Nuclear Density and Morphometry

Samples approximately midway between the base and apex that were immediately adjacent to the LAD (hibernating) and posterior descending arteries (normal) were fixed (10% formalin) and paraffin-embedded. Point-counting of trichrome-stained sections was used to quantify connective tissue [6]. PAS stained sections were used to quantify myocyte diameter. Myocyte diameter was assessed by counting at least 100 cells from the inner and outer half of the LAD and remote regions. Myocytes were included regardless of size as long as myofilaments could be identified surrounding the nucleus. We assessed regional myocyte nuclear density in PAS stained sections as previously described [12]. To evaluate the possibility that changes in nuclear density reflected increases in the number of nuclei per myocyte, longitudinal myocytes (n = 20 in each histological sample) that had boundaries that were clearly visible (intercalated discs and the lateral borders of the myocyte) were used to quantify the number of nuclei per cell in each heart using previously published methodology [17].

#### Immunohistochemical Assessment of Myocyte Proliferation and Angiogenesis

All of the antibodies we employed have been successfully used in the pig in previous studies by our group [12]. Paraffin-fixed tissue sections with 4 µm thickness were incubated with anti-phospho-histone-H3 rabbit polyclonal antibody (Upstate Biotech, 1∶1000) to detect proliferating cells and anti-cTnI (rabbit polyclonal antibody, Santa Cruz, 1∶200) to detect myofilaments. For capillary density evaluation, paraffin-fixed tissue sections were incubated with Von Willebrand factor (vWF, Biocare Medical, 1∶200). Myocardial levels of CD45 negative resident and bone marrow derived progenitor cells (CD45 antibody, 1∶200, AbD serotec) were quantified in frozen tissue sections using the cell surface marker cKit (AbD serotec, 1∶200) and CD133 (Miltenyi biotec, 1∶200) [12], [18]. To optimize identification of cKit and CD133 antigens, we conducted the quantitative analysis using frozen tissue sections. Samples were post-treated with Alexa Fluor 488 conjugated anti-mouse and Alexa Fluor 555 conjugated anti-rabbit antibody (Invitrogen). Nuclei were stained with TO-PRO-3 (Molecular Probes) or DAPI (Vectashield). Image acquisition was performed with a confocal microscope (Zeiss 510 Meta) and AxioImager equipped with ApoTome (Zeiss). Phospho-histone-H3 positive myocytes were counted and evaluated as positive nuclei per million myocyte nuclei as previously described [12]. The number of cKit+ and CD133+ cells in myocardium was also expressed in relation to myocyte nuclear density or cells per million myocytes. Data represent the averages from 462±46 fields examined per slide (area of 72±7 mm^2^ per section).

#### Fluorescence in Situ Hybridization of Sex-mismatched CDC Donor-Recipient Pairs

To track the fate of infused CDCs in the myocardium, male CDCs were injected into female recipients and Fluorescence in Situ Hybridization used to quantify the frequency of Y-chromosomes from donor CDCs (Y-FISH). We hybridized tissue samples with FITC-conjugated porcine Y-chromosome probe (IDLabs, Ontario, Canada) according to the manufacturer’s instructions. The nuclear diameter is greater than the section thickness and thus, the Y chromosome is not present in each nucleus sectioned. We therefore also evaluated the frequency of Y-FISH staining in male control cardiac tissue to determine the efficiency of Y chromosome identification. Tissues were incubated with cTnI, vWF and alpha-SMA to detect co-localization of donor derived Y chromosome cells in myocytes, endothelial cells and vascular smooth muscle. Nuclei were stained with DAPI (Vectashield) or TOPRO3 (Molecular Probes).

#### Flow Cytometry of Circulating Progenitor Cells [12]

We determined whether icCDCs mobilized hematopoietic progenitor cells (cKit+ or CD133+) in hibernating (n = 3) and normal swine (n = 3) as we have previously described. Mononuclear cells were isolated from peripheral blood (30 ml) using the Becton Dickinson CPT cell separation system before, 3 days and 2 weeks after icCDC treatment. Leukocyte counts were performed using an automated hemocytometer while monocyte counts were done using a manual hemocytometer. Approximately 10–20×10^6^ mononuclear cells were analyzed by FACS after staining for cKit (CD117, AbD Serotec), CD133 (PE conjugated, Miltenyi Biotech) and CD 45 (PE-Cy5 conjugated, BD Pharmingen). Isotype controls were used as negative controls. Single stains were also performed to determine quality control and for multi-channel compensation. Data were expressed as progenitor cells (cKit+ and CD45−, CD133+ and CD45−) per million mononuclear cells. All cell counts were corrected for the absolute mononuclear cell count. Immunohistochemical analysis and morphometric analysis of excised tissue is summarized below.

### Statistical Analysis

Data are expressed as the mean ± standard error. Differences in physiological parameters at baseline and after treatment with icCDCs as well as comparisons between the hibernating and normally-perfused remote regions of the same heart were assessed using paired t-tests. Differences among icCDC treated animals and age matched untreated animals were assessed using a two-way ANOVA and the post-hoc Holm-Sidak test (Sigma Stat 3.0). For all comparisons, p<0.05 was considered significant.

## Results

### In Vitro Characterization of Porcine CDCs

**Figure 2:**
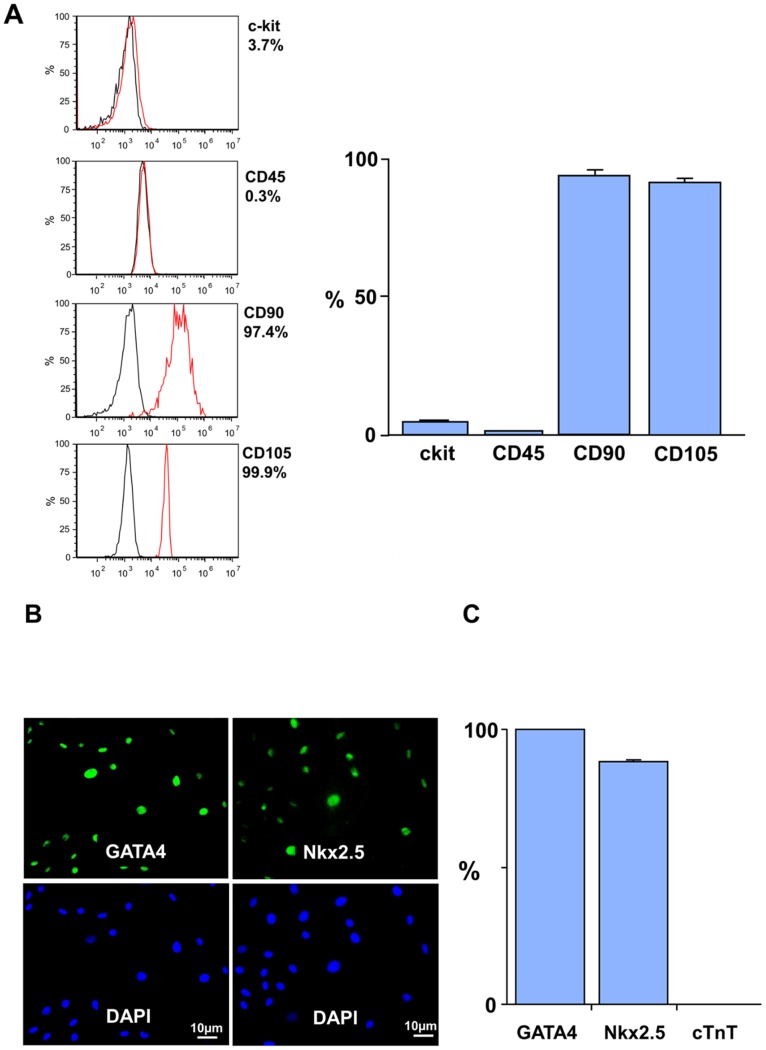
CDC Characterization by Flow Cytometry and Immunohistochemistry. **A.)** Summary of CDC characterization by flow cytometry at passage 6 (n = 6). Most CDCs expressed mesenchymal markers (CD90+ and CD105+) but were CD45 negative. While cKit+ cells were identified in cardiospheres, they declined after plating with 4.7±0.9% of the CDCs remaining cKit positive at the time of icCDC infusion. **B. and C.)** Summary of immunohistochemical characterization of CDCs for markers of commitment to a cardiac lineage (n = 6). At passage 6, most CDCs expressed the cardiac transcription factors GATA4 and Nkx2.5 (green stain). In contrast, CDCs did not express cardiac troponin T, suggesting that these cells are at an early stage of cardiac development.

Porcine cardiospheres were readily isolated from ∼10 mg ventricular needle biopsies. After plating on fibronectin, they expanded to >30 million CDCs after 4–6 passages and 3 to 4 weeks in culture. **Figure 2** summarizes the immunohistochemical and flow cytometry markers of the CDCs at the time of injection. At the time they were infused into swine (passage 6), most CDCs expressed mesenchymal markers (CD90+ and CD105+, **Figure 2A**) but were CD45 negative. The frequency of cKit+ cells declined after plating with 4.7±0.9% of the CDCs remaining cKit+ at passage 6. At this time, virtually all of the CDCs expressed the cardiac transcription factors Nkx2.5 and GATA4 (**Figure 2B**) but they remained cardiac troponin negative. Thus, at the time of intracoronary infusion, our CDCs were primarily a population of early, cardiac-committed progenitors with a low frequency of cKit positivity.

### Safety of Global icCDC Infusion and Quantitative Retention of icCDCs in Normal Swine

**Figure 3:**
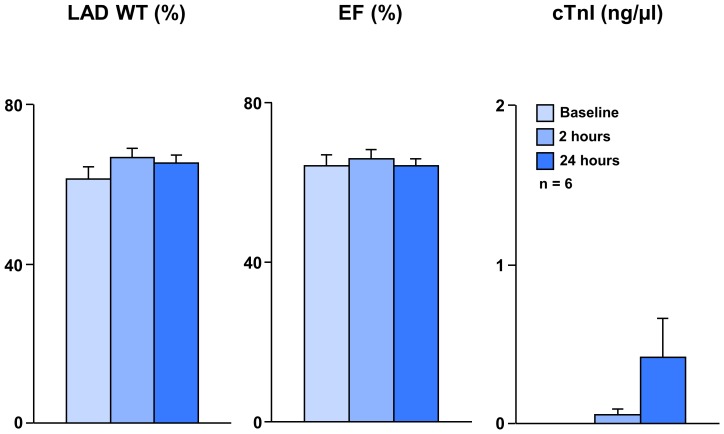
Safety of Global icCDC Infusion under Continuous Flow in Normal Swine. Allogeneic icCDCs (∼38.5±0.9×10^6^ cells, n = 6) were infused into each of the three major coronary arteries and hearts were harvested 24-hours later for pathological analyses. This figure summarizes echocardiographic function and serum TnI at baseline, 2-hours and 24-hours after icCDCs in normal animals. Two hours after icCDCs, there was no change in regional wall thickening (LAD%WT) or global function (EF). Twenty-four hours after icCDCs, function remained unchanged. There was a small statistically insignificant increase in cTnI. Tetrazolium staining and histology showed no evidence of micro-necrosis. Similar results were seen in normal hearts harvested at 2-weeks (data not shown). These results, along with the absence of hemodynamic and electrocardiographic changes during infusion, indicate that global icCDC infusion is safe, has no effect on myocardial function and produces no pathological evidence of infarction.

We initially evaluated the safety of infusing divided doses of allogeneic icCDCs (∼38.5±0.9×10^6^ cells, 18±0.4 µm diameter, n = 6) into each of the three major coronary arteries (nominal rate 1.3×10^6^ icCDCs/minute). Continuous electrocardiographic monitoring demonstrated no ST elevation or arrhythmias during infusion. Echocardiographic evaluation and serum TnI are summarized in **Figure 3**. There was no change in LAD function (LAD % WT 61±7% at baseline to 66±5% at 2-hours, p = ns) and no elevation of cTnI after icCDC infusion (0 to 0.05±0.03 ng/ml at 2-hours, p = ns). When reassessed at 24-hours, function remained unchanged (%LAD wall thickening (%LADWT) 65±2%, p = ns vs. baseline) with a small, insignificant increase in cTnI (0.41±0.24 ng/ml, p = 0.15). There was no TTC evidence of infarction and no histologic increase in connective tissue or light microscopic evidence of necrosis. Thus, global icCDC infusion without employing transient coronary occlusion did not affect myocardial function nor did it produce significant changes in troponin I or pathological evidence of microinfarction.

We quantified retention of CDCs in the heart, by counting myocardial Y-FISH positive cells in female animals 24-hours after male icCDCs (n = 6). There were 5±2 Y-FISH positive cells per million myocytes (1.4±0.5 per cm^2^ section) after icCDCs. Since the efficiency of Y-FISH positivity in male control hearts was 40±4%, the frequency of Y-positive nuclei underestimated actual cell number by a factor of 2.5. Correcting for this factor and extrapolating data to the entire heart indicated that approximately 1.1±0.5 million CDCs or CDC derived cells were present in the heart at 24 hours. This represented 3±1% of the original icCDC dose. This low value is similar to short-term cell retention after intracoronary infusion reported by others using the stop-flow technique as well as after intramyocardial injection[19]–[21]. The number of Y-FISH positive cells (original icCDCs as well as myocardial cells derived from CDCs) remained approximately similar 2-weeks after sex-mismatched icCDC infusion (3.4±0.7% of injected dose, p-ns vs 24-hours). Thus, while viable CDC derived cells remained in the myocardium, there was no significant increase in the number of donor derived cells beyond those present at 24 hours.

### Effects of icCDCs on Myocardial Flow and Function in Swine with Hibernating Myocardium

Baseline physiological studies at 3-months confirmed viable dysfunctional (hibernating) myocardium in all animals (n = 25). At this time there was a severe proximal LAD stenosis (99±1%) with total occlusion and collateral-dependent myocardium in most swine (20 of 25 animals). Regional LAD wall thickening (LAD % WT) was reduced in comparison to normal remote regions (29±3% in LAD vs. 76±7% in remote, p<0.05) with reductions in resting perfusion (LAD 0.83±0.08 vs. 1.32±0.11 ml/min/g in remote, p<0.05). Flow during adenosine vasodilation was severely attenuated (LAD 0.92±0.19 vs. 4.50±0.49 ml/min/g in remote, p<0.01). Although VT/VF develops in the absence of infarction in this animal model of hibernating myocardium [22], survival to the final study was similar in animals after receiving icCDCs (12/15; 80% survival) vs. untreated controls (7/10; 70% survival).

**Figure 4:**
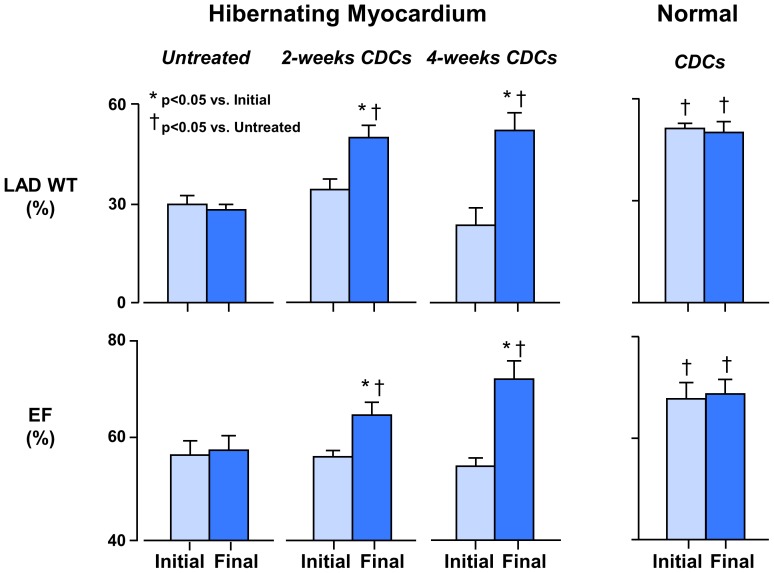
Effects of icCDCs on Regional and Global Function in Hibernating and Normal Hearts. Most animals with hibernating myocardium developed a chronic LAD occlusion in the absence of infarction. Regional LAD wall thickening (% LAD WT) was chronically depressed in hibernating animals as compared to normal remote regions (29±3% vs. 76±7%, p<0.05) with a mild reduction in ejection fraction (EF) vs. normals (57±3% vs. 68±3%, p<0.05). In untreated animals with hibernating myocardium, serial physiological parameters were stable with no change in LAD wall thickening or ejection fraction between studies performed up to 4-weeks later. In contrast, in animals treated with icCDCs, regional LAD wall thickening increased along with a return of ejection fraction to normal. There was no effect of icCDCs on regional or global function in normal animals. Light Blue-Initial; Dark Blue-Final.

**Figure 4** and **Table 1** summarize the serial functional effects of icCDCs. There was no effect of icCDCs on heart rate or blood pressure. In untreated animals, LAD % WT was depressed at rest and did not change when evaluated 4-weeks later (LAD % WT 29.2±2.7% vs. 28.1±1.9%, p = ns) as we have previously demonstrated [12]. In contrast, function after icCDCs significantly increased at 2-weeks (LAD % WT 33.9±3.1% to 49.1±4.0%, p<0.01) and 4-weeks (LAD %WT 22.8±5.6% to 51.0±5.1%, p<0.01). Interestingly, while there was no effect of icCDCs on %WT in normally-perfused remote myocardium after 2-weeks (Remote %WT 85±7% to 80±9%, p = ns), it significantly increased after 4-weeks (Remote %WT 68±10% to 107±11% at 4-weeks, p<0.05). Global function was mildly reduced at rest before icCDCs and returned to values similar to normal animals after icCDC infusion (LVEF 56±1% to 64±2% at 2-weeks and 54±2% to 71±4% 4-weeks after icCDCs, p<0.05).

**Table 1:** Effects of icCDCs on Echocardiographic Measurements of Cardiac Function and Hemodynamic Variables in Swine with Hibernating Myocardium.

	n	LAD Δ WT (mm)	Remote Δ WT (mm)	FS (%)	EF (%)	LV_Sys_ (mm Hg)	HR (bpm)	LVdP/dt_Max_ (mmHg/sec)
***Untreated Hibernating***	7							
Initial		2.8±0.2	6.4±0.7	24.4±1.6	53±2	128±3.0	112±7.5	2171±64
Final		3.2±0.3	7.7±0.7	24.9±1.6	53±3	124±4.0	101±7.6	2035±111
***CDCs Hibernating 2-weeks***	6							
Initial		2.7±0.2	5.8±0.3	29.2±0.9	56±1	138±6.7	110±9.0	2375±89
Final		4.8±0.3*†	6.9±0.5*	34.9±1.7*	64±2*†	139±4.1	107±6.5	2528±74*†
***CDCs Hibernating 4-weeks***	6							
Initial		2.0±0.4	5.2±0.9	26.4±2.2	54±2	144±3.4	86±2.3	2370±97
Final		5.0±0.4*†	8.1±0.8*	35.4±2.9*†	71±4*†	150±3.1	83±5.2	2633±148*†

Values are mean ± SEM; *p<0.05 vs. Initial; **†** vs. Untreated; LAD – Left Anterior Descending Artery; LV – Left Ventricular; WT – Wall Thickening; FS – Fractional Shortening; EF – Ejection Fraction; Δ WT = End-Systolic Wall Thickness – End-diastolic Wall Thickness.

**Figure 5:**
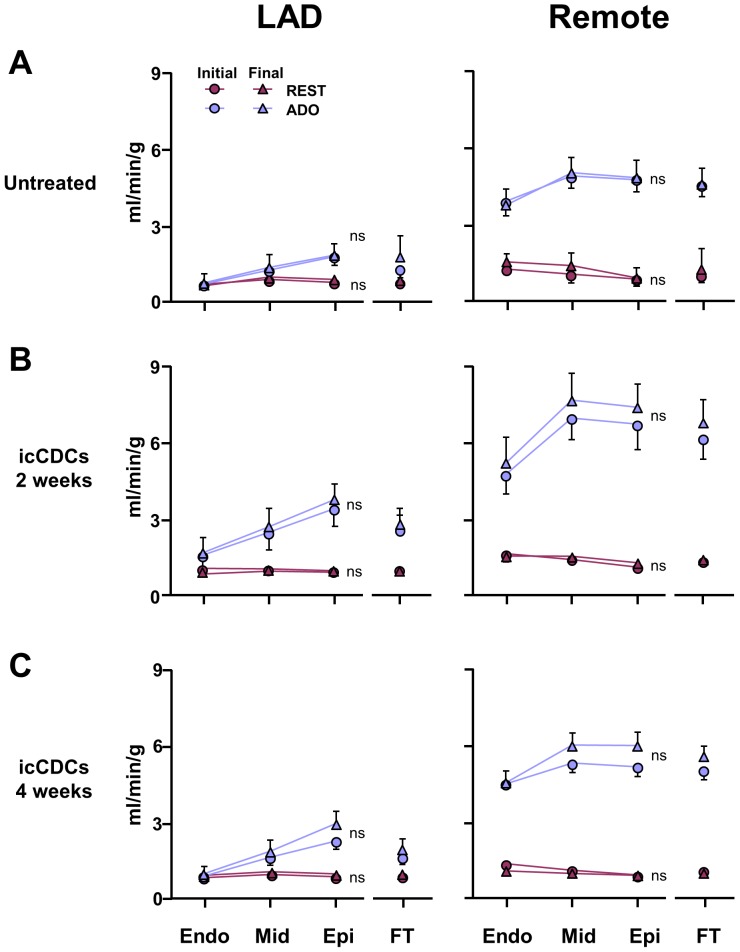
Administration of icCDCs Does Not Alter Myocardial Perfusion. Serial microsphere measurements of transmural myocardial perfusion at rest (solid symbols) and pharmacological vasodilation with adenosine (open symbols) in swine with hibernating myocardium. At the initial baseline study, coronary flow reserve was critically impaired in the LAD region but increased over 4-fold after adenosine in normally perfused remote regions. In untreated animals (**A**), paired analysis of initial and final measurements demonstrated stable myocardial perfusion indicating no spontaneous improvement in coronary collateral flow in this model over time. Paired analysis of initial and final measurements of coronary flow at rest and vasodilation also did not change either 2-weeks (B) or 4-weeks (C) after icCDC infusion. These results indicate that the functional improvement seen after icCDC infusion is not related to an increase in coronary collateral perfusion in swine with hibernating myocardium.

**Figure 6:**
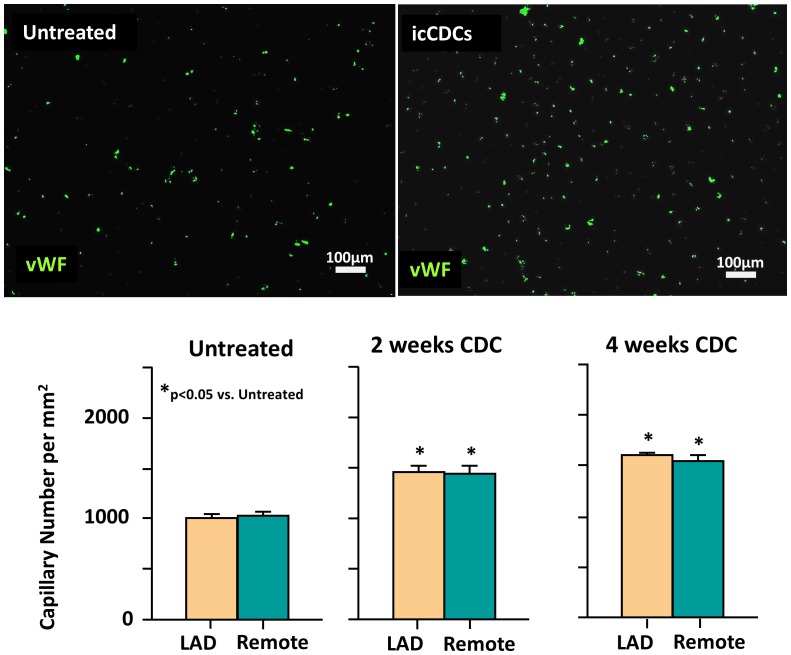
icCDCs Increased Capillary Density in Hibernating Myocardium. Capillary density was quantified using von Willebrand Factor (vWF). Upper images show vWF staining (green) from hibernating myocardium. Animals receiving icCDCs exhibited increased capillary density at 2-weeks and 4-weeks vs. untreated animals with hibernating myocardium (both p<0.05 vs. untreated, lower graph). While icCDCs stimulated capillary angiogenesis, their contribution to coronary vascular resistance was small since there was no functional improvement in coronary collateral perfusion at rest or after pharmacological vasodilation with adenosine. This is consistent with the negligible contribution of the low resistance capillary bed to total coronary vascular resistance.

Despite the improvement in function in hibernating myocardium, there was no significant effect of icCDCs on paired serial measurements of coronary flow at rest or during adenosine vasodilation (**Figure 5**). As a result, while LAD flow reserve (adenosine/rest) was critically impaired in hibernating myocardium, it did not increase significantly after icCDCs (LAD adenosine/rest 1.88±0.20 initial vs. 2.03±0.31 final, p = ns). Coronary flow reserve also remained unchanged in normally-perfused remote myocardium (4.9±0.5 initial vs. 5.6±0.6 final, p = ns). Likewise, serial measurements of relative flow during adenosine vasodilation (LAD/remote) remained unchanged after icCDCs (0.18±0.04 initial vs. 0.19±0.08 final, p = ns). While there was no measurable increase in coronary collateral flow, icCDCs stimulated capillary angiogenesis and increased myocardial capillary density (1013±31/mm^2^ in untreated animals to 1604±27/mm^2^ at 4-weeks after icCDCs, p<0.05 vs. untreated, **Figure 6**). Thus, improvements in regional and global function after icCDCs were not secondary to stimulation of increased coronary collateral flow or arteriogenesis.

### Effects of icCDCs on Myocyte Proliferation

**Figure 7:**
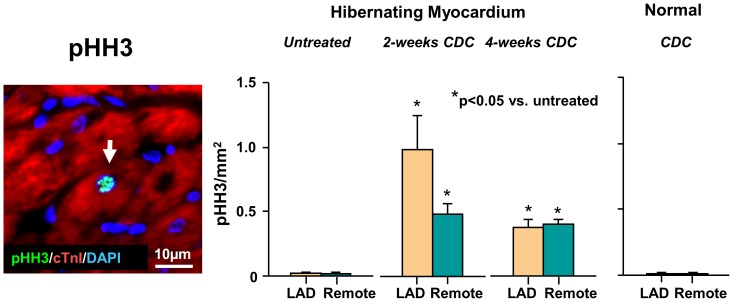
Effects of icCDCs on Cardiomyocyte Markers of Mitosis. Mitotic myocytes were quantified with phospho-histone-H3 (arrow, pHH3 positive myocyte nucleus localized with co-staining for DAPI and TnI). We found that icCDCs increased myocytes in the mitotic phase of the cell cycle in hibernating LAD and remote regions 2-weeks after injection. These declined but remained significantly elevated as compared to untreated animals and normal controls 4-weeks after icCDCs. There was no effect of icCDCs on the number of pHH3 positive myocytes in normal animals.

**Figure 8:**
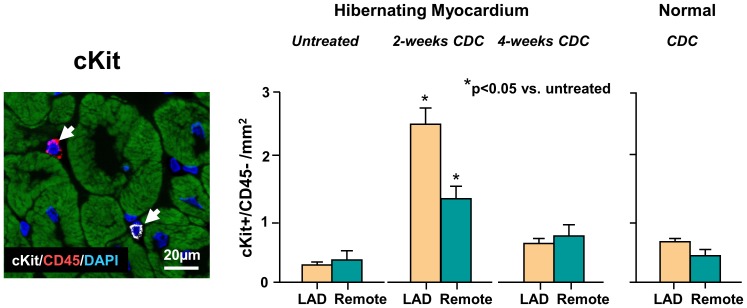
Effects of icCDCs on Myocardial cKit Cells. cKit positive cardiac progenitor cells (white) that did not express CD45(red) were counted and quantified in each heart. Intracoronary CDCs produced a transient increase in cKit+/CD45- cells at 2-weeks which returned to baseline after 4-weeks. Increases in cKit+ cells were global and similar in dysfunctional LAD and remote regions of hibernating hearts. In contrast, there was no effect of icCDCs on cKit+ cells in normal hearts. LAD-yellow bars; Remote-green bars.

Intracoronary CDC infusion increased myocytes in the mitotic phase of the cell cycle throughout the entire hibernating heart. **Figure 7** shows that phospho-histone-H3 (pHH3) positive myocytes increased in both LAD and remote regions of hibernating hearts and remained significantly elevated at 4-weeks. In contrast, despite a significant retention of icCDCs in normal control hearts, they did not stimulate myocyte mitosis and pHH3 positive myocytes remained low and similar to untreated normal animals. These data indicate that icCDCs stimulated myocyte proliferation in ischemic as well as remote myocardium of the diseased heart with no effect on myocyte proliferation in the normal heart. **Figure 8** summarizes the effects of icCDCs on resident myocardial cKit^+^ cells. Rare cKit+ cells were identified in untreated animals with hibernating myocardium as well as normals. Total cKit^+^ cells (as well as cKit^+^/CD45^−^ cells) increased from 0.8±0.2/mm^2^ (cKit^+^/CD45^−^: 0.2±0.04/mm^2^) in untreated animals to 4.8±0.3/mm^2^ (cKit^+^/CD45^−^: 2.5±0.1/mm^2^) at 2 weeks after icCDCs (both p<0.05 vs. untreated). The number remained elevated at 2.7±0.3/mm^2^ (cKit^+^/CD45−: 0.7±0.1/mm^2^, p<0.05 vs. untreated) at 4 weeks after icCDCs. In contrast, icCDCs did not increase total cKit^+^ (1.3±0.2/mm^2^, p-ns vs. untreated) or cKit^+^/CD45^−^ cells (0.8±0.1/mm^2^, p-ns vs. untreated) in normal animals.

**Figure 9:**
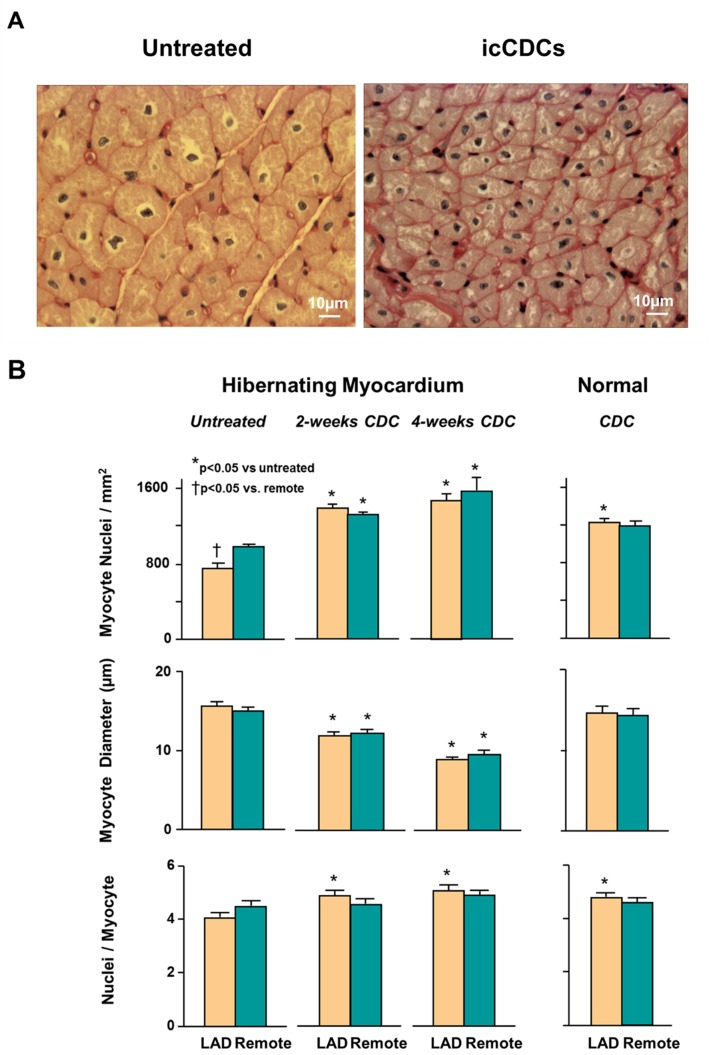
Quantitative Effects of icCDCs on Myocyte Nuclear Density and Myocyte Diameter in Dysfunctional LAD Regions and Normally-Perfused Remote Myocardium. **A.)** PAS staining demonstrates the typical increase in myocyte nuclear density and reduction of myocyte size found in a dysfunctional LAD region after icCDC treatment. Myocyte diameter in untreated hibernating myocardium was increased due to the loss of myocytes arising from apoptosis during the development of hibernating myocardium as we have previously described [6]. **B).** After icCDC infusion, there was a progressive increase in LAD myocyte nuclear density and a reduction in myocyte diameter that was significant as early as 2-weeks after treatment. Directionally similar changes were observed in remote regions from hibernating hearts. There was no effect of icCDCs on myocyte nuclear density or myocyte diameter in normal animals. Evaluation of longitudinally oriented myocytes showed only small differences in the number of nuclei per myocyte. While values of nuclei per myocyte were lower in untreated hibernating myocardium, they rose to become no different than normal hearts after icCDCs. LAD-yellow bars; Remote-green bars.

Since cell cycle markers such as pHH3 measured at a single time point cannot quantify the cumulative number of new myocytes produced, we quantified myocyte nuclear density, nuclear number per myocyte and myocyte diameter from histological sections in animals with and without treatment with icCDCs [12]. Concordant with the global increase in the mitotic marker pHH3, we found that icCDCs increased myocyte nuclear density in LAD as well as remote regions of the hibernating heart. At the same time, cardiomyocyte size was substantially smaller after icCDC infusion (**Figure 9**). Myocyte diameter decreased from 15.7±0.4 to 8.9±0.3 µm in the hibernating LAD region (p<0.05) and from 15.0±0.4 to 9.5±0.4 µm in remote myocardium (p<0.05). The myocyte diamters after icCDCs were also notably smaller than myocyte size in our previous publications of untreated animals with or without hibernating myocardium [12], [18], [23]. At the same time, LAD myocyte nuclear density which was reduced in the hibernating region in untreated animals (749±56 myocyte nuclei/mm^2^) increased to 1472±74 after icCDCs (p<0.05). Likewise, it rose from 981±22 to 1574±145 myocyte nuclei/mm^2^ in the remote region (p<0.05). Despite increases in the number and reductions in the size of myocytes after icCDCs, gross pathological analysis confirmed that icCDCs did not increase global LV mass (icCDC treated 2.0±0.2 vs. 2.4±0.4 g/Kg by postmortem analysis, p-ns)**.** Consistent with the lack of effect of icCDCs on mitotic markers in normal hearts, there was no effect of icCDC infusion on myocyte nuclear density or myocyte diameter in normal controls. Interestingly, icCDCs also attenuated myocardial interstitial fibrosis. Interstitial connective tissue in untreated hibernating LAD regions was 6.6±1.4% vs. 4.1±0.8% in remote regions. After icCDCs, LAD connective tissue decreased similar to remote regions (3.4±0.3% vs. 3.7±0.1%, p-ns vs. untreated hibernating myocardium).

To exclude the possibility that icCDCs simply stimulated myocyte nuclear division (with increased nuclear number per cell) without cell division, we assessed average myocyte nuclear number per cell in longitudinally-oriented myocytes in each histological sample (**Figure 9B**). Porcine myocytes in normal hearts were multi-nucleated with 4.8±0.1 nuclei/myocyte, similar to values obtained in isolated porcine myocytes [24]. There was a small reduction in nuclei/myocyte from animals with untreated hibernating myocardium (LAD region 4.1±0.1 nuclei/cell, p<0.05 vs. normal animals) while remote regions remained unchanged (4.5±0.2 nuclei/myocyte vs. 4.6±0.1 nuclei/myocyte in normal animals, p-ns). Four-weeks after global icCDC infusion, the number of nuclei/myocyte in hibernating myocardium increased modestly to values similar to those found in normal hearts (5.0±0.2 nuclei/myocyte in LAD and 4.8±0.2 nuclei/myocyte in remote after icCDCs; p-ns vs. normal hearts). These data indicate that some of the increase in myocyte nuclear density after icCDCs reflected normalization of the reduced nuclear number/myocyte that was present in hibernating myocardium. Nevertheless, the relative increase in nuclei per cell were smaller than the measured increase in myocyte nuclei per mm^2^ after icCDCs.

### Estimate of Myocytes Derived Directly From icCDCs vs. Endogenous Myocyte Regeneration

**Figure 10:**
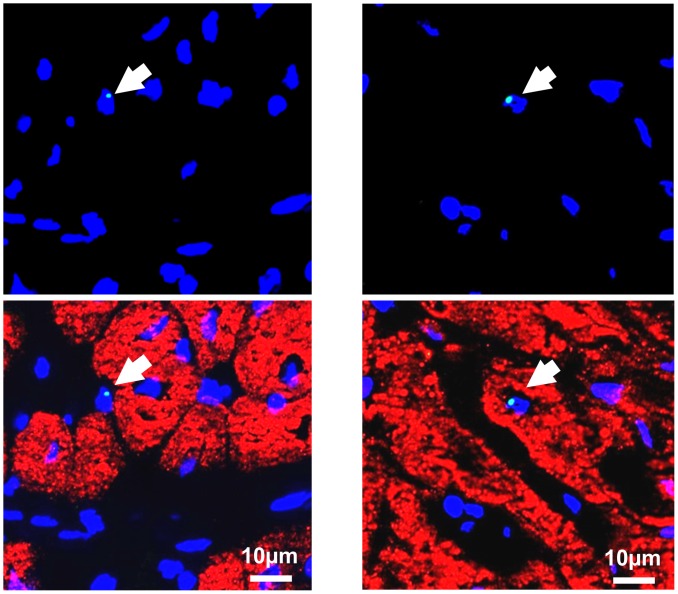
Examples of Sex-Mismatched CDC-Derived Cells in Hibernating Myocardium. To determine the fate of injected CDCs, male donor CDCs were injected into female recipients and Y-chromosomes were detected by fluorescence in situ hybridization (Y-FISH). An average of 3.4% of the total injected CDC dose wad retained in the left ventricle at 2-weeks. Y-chromosome positive cells were primarily found in the interstitial space. The arrows in the pair of photomicrographs in the left upper and lower panels demonstrate a Y-chromosome nucleus (green) in an interstitial cell with the nucleus stained blue (DAPI). This is distinct from the cardiac myocytes which are stained with troponin I in red. While infrequent, we identified rare Y-positive cardiac myocytes as depicted by the arrow on the upper and lower right panel. This indicates the potential for CDCs to differentiate into myocytes. Nevertheless, these constituted less than 1 in 5,000 of the new myocytes formed. None of the Y-FISH positive cells co-localized with cKit.

To determine the number of new myocytes that were directly derived from icCDCs, we assessed Y-FISH positive cells in the hearts of female animals treated with male donor icCDCS 2-weeks after treatment (n = 5). We found 7±1 Y-FISH positive cells per million myocyte nuclei after icCDCs (1±0.1 per cm^2^ tissue section). Importantly, this was two orders of magnitude lower than the frequency of proliferating myocytes (∼600 pHH3 positive myocytes per million myocytes or ∼98 per cm^2^, **Figure 7**). When these measurements were extrapolated to the entire heart, only ∼3.4±0.7% of the original icCDC dose (or a total of∼1 million CDCs or CDC-derived cells) remained in the heart. Most donor cells were non-myocytes but there were rare examples of Y-FISH positive cardiomyocytes derived from donor CDCs (**Figure 10**). Specifically, 1.0±0.7 Y-FISH positive cells per million myocyte nuclei were also positive for cardiac troponin I, indicating that ∼15% of the retained CDCs differentiated into cardiomyocytes. Even if we assumed that each Y-FISH positive cell will differentiate into a cardiomyocyte, CDC-derived myocytes would represent no more than 1 in every 5000 myocytes (or 0.05%). Thus, the paucity of Y-FISH staining in the face of significant increases in myocyte nuclear density indicates that most of the new myocytes arose from the recipient and not via differentiation of donor CDCs.

### Effects of icCDCs on Circulating cKit^+^ and CD133^+^ Bone Marrow Progenitor Cells (n = 6)

Since CDCs expressed mesenchymal markers and we previously demonstrated that intracoronary mesenchymal stem cell infusion mobilizes bone marrow progenitor cells which contribute to cardiac repair [12], we evaluated circulating progenitor cells after icCDCs. FACS analysis demonstrated that icCDCs did not increase circulating cKit^+^ or CD133^+^ cells when assessed either 3-days or 2-weeks after icCDC infusion. Since the results were similar in hibernating and sham animals, the final results were pooled. Bone marrow mononuclear cells that were cKit+ and CD45- averaged 6.9±1.7% at baseline, 3.2±0.2% 3-days after icCDCs and 4.2±1.5% 2-weeks after icCDCs (all p-ns vs. initial baseline values). The percentage of bone marrow mononuclear cells that were CD133+ but negative for hematopoietic lineage markers (CD45−) averaged 0.02±0.01%, 0.02±0.01% at 3 days and 0.06±0.02% after 2-weeks (all p-ns). Circulating mononuclear cells were not affected by icCDCs with cKit+/CD45− cells averaging 1.2±0.3% at baseline, 0.3±0.04% after 3-days and 1.2±0.4% after 2-weeks (all p-ns). Likewise, circulating CD133+/CD45- mononuclear cells averaged 0.05±0.02% at baseline, 0.02±0.01% after 3-days and 0.06±0.03% after 2-weeks (all p-ns), and CD133+ cells were rarely observed in myocardial tissue after icCDCs (data not shown). Thus, unlike the intracoronary infusion of autologous mesenchymal stem cells [12], icCDCs did not mobilize bone marrow progenitor cells.

## Discussion

Our results demonstrate that global infusion of icCDCs without using the “stop-flow” technique is a feasible, safe and efficacious approach to administer CDCs to the entire heart. Allogeneic icCDCs given with cyclosporine immunosuppression improved global and regional contractile function in swine with hibernating myocardium with no effect when administered to normal controls. This improvement was accompanied by increased myocyte nuclear density and a reduction in myocyte size in the dysfunctional hibernating region as well as in normally-perfused remote regions of the hibernating heart. New myocytes were primarily derived from endogenous sources since Y-FISH staining demonstrated only rare Y-FISH positive myocytes. Collectively, our results in an animal model with contractile dysfunction that is devoid of infarction support the feasibility of employing global intracoronary infusion of allogeneic CDCs to treat viable dysfunctional myocardium in patients with chronic ischemic heart disease.

### Relation to Previous Studies

Autologous icCDCs have previously been infused into the infarct-related artery using the “stop-flow” technique. Using this approach, studies in swine [10] and the Phase I/II CADUCEUS trial [5] confirmed that CDCs regenerate muscle mass and decrease scar volume yet global systolic function assessed from the ejection fraction did not increase. Our findings raise the possibility that this may relate to restricting CDC delivery to the infarct zone. As a result, deleterious myocyte loss and dysfunction in the large amount of remodeled myocardial tissue remote from the infarct is not effectively treated [7], [8]. With intracoronary infusion under continuous flow, the percentage of injected CDCs residing in the heart at 2-weeks was similar to intracoronary MSCs [12], [25] and similar to the CDC retention reported by others using “stop-flow” or direct intramyocardial injection[21], [26]–[28]. At the same time, the troponin I levels we found after global intracoronary infusion were lower than many studies using the “stop-flow” technique to administer cell-therapies into the infarct-related artery [5], [10], [29], [30]. This could be due to the use of a Millipore filter, a lower regional cell dose or preventing in vivo aggregation of cells by infusing them under flow. Based on these findings, global CDC infusion appears to be a safe and clinically relevant alternative approach for intracoronary stem cell delivery. Further clinical studies will be required to establish feasibility in patients.

### Source of New Myocytes After icCDCs

We found that icCDCs administered to the entire heart stimulated myocyte proliferation leading to an increase in myocyte nuclear density in a fairly short period of time. At the same time, there was a marked reduction in myocyte size throughout the entire heart. These changes were associated with an elevation in pHH3-positive myocytes indicative of myocyte cell division. Our results indicate that allogeneic icCDCs primarily facilitate endogenous myocyte proliferation of host recipient cells since the frequency of Y-FISH donor cells (including myocytes and non-myocytes) in tissue harvested at postmortem was extremely low. In addition, Y-FISH positive cells were also much lower than myocardial cKit+ cells suggesting that most tissue cKit+ cells likely arose from the host rather than donor CDC population. Although we [12] and others [31] have used cardiac localization of cKit+ cells as a marker of endogenous cardiac stem cell mobilization, it is important to note that the role of endogenous cKit+ cells in forming cardiomyocytes has recently been questioned [32]. Based upon our findings, the majority of newly formed myocytes probably arose from endogenous myocyte cell division but we cannot exclude a contribution arising from the differentiation of endogenous cardiac stem cells since we are unable to discriminate between these two possibilities in the large animal model. Support for both mechanisms is provided by a recent study documenting an approximately equal contribution of cardiomyocyte proliferation and endogenous stem cell recruitment to new myocyte formation following CDC treatment in mice with myocardial infarction [33].

Regardless of the cellular source of new myocytes, the specific mechanism(s) by which CDCs stimulate endogenous myocyte regeneration remain elusive. Though not tested in the present study, one possibility is that icCDCs synthesize and release growth factors that stimulate endogenous myocyte proliferation. A similar mechanism has been proposed for MSCs [12]. Alternatively, it is plausible that icCDCs interact with resident CSCs or myocytes and effect cell-cell transfer of microRNAs or exosomes that allow myocytes or CSCs to re-enter the proliferative phase of the cell cycle [34]. Recent work in small animal models of myocardial infarction supports this notion. For example, Xie et al. demonstrated that β1 integrin signaling mediates cell-to-cell contact-dependent CDC stimulation of myocyte proliferation [35], while Ibrahim et al. identified secretion of microRNA-rich exosomes as a key mediator of CDC-mediated cardiac regeneration [36]. This exciting progress may facilitate the future development of new strategies to promote myocardial repair without the use of exogenous cells.

### Autologous vs. Allogeneic icCDCs

While most previous studies in swine have used autologous CDC preparations [10], [37], we found that allogeneic icCDCs were effective at stimulating myocyte proliferation and improving function when given with cyclosporine immunosuppression. Others have recently demonstrated the efficacy of allogeneic CDCs in rodents and more recently confirmed this in swine without using cyclosporine immunosuppression [21], [38]. This likely reflects the fact that CDCs are immunoprivileged and do not express class II MHC molecules. It is also compatible with the notion that, like intracoronary mesenchymal stem cells (icMSCs) [12], CDCs facilitate endogenous cardiac regeneration and are not the direct precursor of most of the myocytes formed. Recently, Malliaras and colleagues demonstrated that myocyte regeneration after CDC treatment arises primarily from the host tissue using genetic fate mapping in mice [33] and histological evaluation in a swine model of myocardial infarction [21]. Our results provide additional support for this mechanism in a large animal model of ischemic LV dysfunction without infarction and confirm the feasibility of using allogeneic CDCs with cyclosporine to promote cardiac repair. Although allogeneic CDCs are effective without immunosuppression [21], [38], cyclosporine may have enhanced their effects by delaying an immune response to other mismatched donor antigens. Nevertheless, based upon the fact that most of the myocyte regeneration appears to be derived via recipient myocyte proliferation rather than derived from the donor CDCs in our sex mismatched recipients, it seems unlikely that chronic cyclosporine would be necessary once endogenous repair has been stimulated. While further studies could determine whether cyclosporine affords any advantage over using allogeneic CDCs alone, allogeneic icCDC therapy using the stop-flow approach and regional infusion to the infarct related artery without immunosuppression has recently been advanced to a Phase I/II clinical safety trial (NCT01458405, ClinicalTrials.gov).

### Comparison of the Effects of icCDCs and icMSCs on Myocyte Regeneration

The effects of allogeneic icCDCs on myocyte regeneration are qualitatively similar to changes we reported after autologous icMSCs in swine with hibernating myocardium [12]. The two cell types are somewhat similar in that they each express mesenchymal surface antigens such as CD90 and CD105 and stimulate myocyte proliferation throughout hearts with viable dysfunctional myocardium. Nevertheless, they differ in other respects and the cellular mechanisms responsible for cardiac repair may differ. For example, although both icCDCs and icMSCs increased myocardial cKit+ cells and increased myocytes in the mitotic phase of the cell cycle, only icMSC treatment resulted in increased myocardial CD133+ bone marrow progenitor cells. Another difference is that porcine icMSCs did not express cKit, although it has recently been argued that the cKit fraction does not contribute to the regenerative efficacy of CDCs [39]. Finally, icMSCs rarely express cardiac transcription factors (GATA4 and Nkx2.5) that were present in nearly all of the icCDCs we injected in the present study. Thus, while both intracoronary stem cell formulations stimulated myocyte regeneration, the precise molecular mechanisms along with the source of new myocytes could differ as could their quantitative impact on myocardial function and the number of new myocytes regenerated. Both autologous MSCs and CDCs have completed early phase I/II clinical trials using approaches that restrict administration to the infarct related artery or employ intramyocardial injection [40]. Since it will be challenging to compare different stem cell therapies in clinical trials, blinded head-to-head comparisons of stem cell formulations in preclinical studies like ours may provide insight into whether one approach is superior to the other to better inform selection of the best clinical platform for cardiac repair.

### Experimental Considerations

In contrast to previous studies examining human CDCs [2], [41] the porcine CDCs used in the present study exhibited very high expression of CD90. This is a consistent finding in our laboratory, as we reliably observe ∼95–99% CD90^+^ expression in porcine CDCs between passage 4 and passage 6. It is unclear if this reflects species-related differences in the antigenic profile of CDCs, but this notion is supported by the fact that our cultivation protocol is identical to that utilized in previously published studies [2], [5]. This may contribute to the paucity of myocytes derived directly from injected CDCs in light of recent *in vitro* data indicating that the CD90− fraction of human CDCs show superior cardiogenic differentiation potential [42]. Nevertheless, our results demonstrate that icCDC-mediated functional cardiac repair is not dependent on the presence of a significant population of CD90^−^ cells. The use of thin (4 µm) tissue sections for immunohistochemical analyses precludes z-stack confocal imaging to confirm the localization of pHH3 positive and Y-FISH positive nuclei but also reduces the likelihood that a non-myocyte nucleus could be superimposed on top of a myocyte. Thus, we believe that the relative changes in pHH3 positive and Y-FISH positive myocytes between untreated and icCDC-treated animals are indicative of increased myocyte proliferation and CDC engraftment, respectively. Finally, it is important to acknowledge the limitations of two-dimensional trans-thoracic echocardiography in providing precise quantification of LV ejection fraction in swine. Future studies should include advanced, three-dimensional imaging approaches (i.e., multi-detector computed tomography or cardiac magnetic resonance imaging), particularly when indices of global LV function are a primary endpoint.

### Translational Implications

The results of the present study suggest that global intracoronary infusion of allogeneic CDCs to the entire heart is safe and efficacious without employing the “stop-flow” technique. Like allogeneic MSCs, allogeneic CDCs would provide an “off-the-shelf” formulation to treat patients with LV dysfunction and myocardial infarction circumventing the need for autologous stem cell harvesting and expansion. This could provide an approach that is easily implemented in any standard catheterization laboratory and broadly available since it would not require advanced instrumentation to inject cells into the myocardium, nor a preexisting coronary stent which is required for the “stop-flow” approach. While further studies are required to determine the most efficacious cell type, the global intracoronary infusion of allogeneic CDCs affords promise as a treatment for regional and global LV dysfunction due to ischemic and non-ischemic LV dysfunction.
